# The Impact of *Schistosoma japonicum* Infection and Treatment on Ultrasound-Detectable Morbidity: A Five-Year Cohort Study in Southwest China

**DOI:** 10.1371/journal.pntd.0000685

**Published:** 2010-05-18

**Authors:** Elizabeth J. Carlton, Michelle Hsiang, Yi Zhang, Sarah Johnson, Alan Hubbard, Robert C. Spear

**Affiliations:** 1 Department of Environmental Health Sciences, University of California, Berkeley, California, United States of America; 2 Department of Pediatrics, University of California San Francisco, San Francisco, California, United States of America; 3 Institute for Parasitic Diseases, Sichuan Center for Disease Control and Prevention, Chengdu, China; 4 Department of Biostatistics, University of California, Berkeley, California, United States of America; Imperial College Faculty of Medicine, United Kingdom

## Abstract

**Background:**

Ultrasonography allows for non-invasive examination of the liver and spleen and can further our understanding of schistosomiasis morbidity.

**Methodology/Principal Findings:**

We followed 578 people in Southwest China for up to five years. Participants were tested for *Schistosoma japonicum* infection in stool and seven standard measures of the liver and spleen were obtained using ultrasound to evaluate the relationship between schistosomiasis infection and ultrasound-detectable pathology, and the impact of targeted treatment on morbidity. Parenchymal fibrosis, a network pattern of the liver unique to *S. japonicum*, was associated with infection at the time of ultrasound (OR 1.40, 95% CI: 1.03–1.90) and infection intensity (test for trend, p = 0.002), adjusting for age, sex and year, and more strongly associated with prior infection status and intensity (adjusted OR 1.84, 95% CI: 1.30–2.60; test for trend: p<0.001 respectively), despite prompt treatment of infections. While declines in parenchymal fibrosis over time were statistically significant, only 28% of individuals with severe parenchymal fibrosis (grades 2 or 3) at enrollment reversed to normal or grade 1 within five years. Other liver abnormalities were less consistently associated with *S. japonicum* infection.

**Conclusions/Significance:**

Parenchymal fibrosis is an appropriate measure of *S. japonicum* morbidity and can document reductions in disease following control efforts. Other ultrasound measures may have limited epidemiological value in regions with similar infection levels. Because severe fibrosis may not reverse quickly following treatment, efforts to reduce exposure to *S. japonicum* should be considered in combination with treatment to prevent schistosomiasis morbidity.

## Author Summary

Schistosomiasis is a water-borne parasite that infects approximately 200 million people worldwide. *Schistosoma japonicum*, found in Asia, causes disease by releasing eggs in the liver, leading to fibrosis, anemia, and, in children, impaired growth. Ultrasound can assess liver pathology from schistosomiasis; however more information is needed to evaluate the relevance of standard ultrasound measures. We followed 578 people for up to five years, testing for schistosomiasis infection and conducting ultrasound examinations to assess the relationship between infection and seven ultrasound measures and to evaluate the impact of treatment with anti-schistosomiasis chemotherapy (praziquantel) on morbidity. All infections were promptly treated. Fibrosis of the liver parenchyma, pathology unique to *S. japonicum*, was associated with schistosomiasis infection, and was most advanced in people with high worm burdens. Liver fibrosis declined significantly following treatment, but reversal of severe liver fibrosis was rare. Other ultrasound measures were not consistently related to schistosomiasis infection or treatment. These findings suggest parenchymal fibrosis can be used to measure morbidity attributable to *S. japonicum* and evaluate the impact of disease control efforts. Because reversal of severe fibrosis was limited, disease control efforts will be most effective if they can not only treat existing infections but also prevent new infections.

## Introduction

Schistosomiasis causes morbidity in the human host through the schistosome egg, which triggers inflammation and fibrosis that can lead to anemia, impaired growth and in severe cases, gastrointestinal bleeding and death [1]–[4]. The major intestinal schistosomes, *Schistosoma japonicum*, found in Asia, and *S. mansoni*, found in Africa, the Americas and the Middle East, mature, mate and lay eggs in the portal and mesenteric blood vessels. Eggs are transported to the liver where they are encapsulated and the granulomas that form induce an inflammatory cascade that includes the deposition of collagen and extracellular matrix proteins, a normal liver repair process that can lead to fibrosis when fibrogenesis exceeds the replacement of scar tissue with healthy cells [5], [6]. The immune regulation of this process is currently being explored [7]. Approximately 700,000 people are infected with *S. japonicum* in China [8]. As in other parts of the world, schistosomiasis control efforts have focused primarily on the distribution of the antischistosomal drug, praziquantel [9], [10]. However the success of such efforts hinges on the ability to reduce not only schistosomiasis infections, but also morbidity. A means of documenting *S. japonicum* morbidity is essential to the evaluation of disease control efforts [11].

Ultrasound is a non-invasive method that can be used to evaluate fibrosis resulting from schistosomiasis infection. Fibrosis along the portal vein and its branches produces a clay pipestem pattern, as the portal tracks are lined with fibrous tissue, and is observed following both *S. mansoni* and *S. japonicum* infection. This periportal fibrosis can be assessed qualitatively through image classification [12] or quantitatively by measuring the diameter of three secondary portal branches [13]. Unique to *S. japonicum* infection is parenchymal fibrosis, a network pattern that is often described as fish scale or tortoise shell-like. Parenchymal fibrosis is likely due to the smaller *S. japonicum* egg size which allows the parasite eggs to enter smaller portal veins and reach a greater portion of the liver [7]. *S. japonicum* adult females produce ten times more eggs per day than *S. mansoni*, and the eggs often are deposited in clusters, two additional factors that exacerbate the severity of *S. japonicum* morbidity relative to other schistosome species and may contribute to the unique fibrotic pattern [3]. Ultrasound can also be used to assess hepatomegaly, splenomegaly and dilation of the portal vein, all of which result from portal hypertension. Recognizing the potential of ultrasound to be used to evaluate the impact of disease control efforts, draft protocols for the use of ultrasound in assessing schistosomiasis morbidity were established for each of the three major schistosome species in 1990 [13], [14]. Follow-up meetings to refine the protocols for *S. haematobium* and *S. mansoni* were held in 1996 and 1997 [15]. Ultrasound is considered the gold standard for schistosomiasis morbidity assessment for these species [11]. However, due to insufficient evidence the protocol for *S. japonicum* has not been revised.

To date, a comprehensive evaluation of the *S. japonicum* ultrasound measures proposed in Cairo, including their relationship to *S. japonicum* infection and the way in which they change following treatment, is lacking. Li et al. [16], [17] offer the most complete examination of the proposed ultrasound measures to date, tracking a highly exposed cohort over five years, but as standard organ sizes have only recently been published [18], assessments of liver and spleen enlargement did not account for participant height as currently recommended [15]. Failure to account for height-specific variation in organ sizes can lead to underestimates of morbidity, particularly in pediatric populations [19]. Ideally, ultrasound measures are associated with *S. japonicum* infection and decline following treatment [13]. The available data is conflicting on both criteria. Treatment led to declines in parenchymal and periportal fibrosis in a highly exposed Chinese cohort; however 68% of participants experienced no change in parenchymal fibrosis over a five-year period [16], [17] and other studies have not found significant declines in periportal fibrosis following *S. japonicum* treatment [20], [21]. Further, a direct relationship between infection and fibrosis or measures of portal hypertension has not been demonstrated consistently [16], [17], [22].

We followed 578 individuals over five years in order to examine the relationship between *S. japonicum* infection and five liver ultrasound measurements recommended in the draft protocol as well as two spleen ultrasound measurements included in the standard Chinese examination. Specifically, we hypothesized that *S. japonicum* infection is associated with ultrasound-detectable measures of hepatic fibrosis and that treatment of infected individuals leads to declines in ultrasound-detected morbidity.

## Methods

Participants were drawn from a cross-sectional survey in 2000 that revealed high infection prevalence (ranging from 3% to 73%) in 20 villages distributed in the hilly terrain of Sichuan Province in Southwest China where irrigated agriculture for the cultivation of rice, wheat, corn and tobacco is the primary source of *S. japonicum* infection [23]. As part of the survey, all residents aged 4 to 60 years in 20 villages Xichang County, Sichuan Province, China were invited to be tested for *S. japonicum* infection and to answer a brief questionnaire. We used a random number generator to select approximately 30% of the population, stratified by village and occupation, for ultrasound examination. Ten villages with high infection prevalence in 2000 were selected to be followed longitudinally using infection surveys and ultrasound examinations over the next five years. Participants in our cohort included individuals who had an ultrasound examination in 2000 and lived in one of the ten villages followed through 2005.

### Ethics statement

All participants provided oral informed consent and were provided treatment following *S. japonicum* positive stool examinations. As the survey procedures used in this study are the same as those used by the Institute of Parasitic Diseases, Sichuan Center for Disease Control and Prevention (IPD) for schistosomiasis surveillance and given the high rates of illiteracy in the population, oral informed consent was obtained and documented by IPD staff. The research protocol and consent procedures were approved by the Sichuan Institutional Review Board and the University of California, Berkeley Committee for the Protection of Human Subjects.

### Ultrasound examination

Ultrasound examinations were conducted in fall 2000, 2002 and 2005. All examinations were conducted by one trained ultrasonographer (YZ) using a single portable ultrasound machine (Hitachi EUB 405, Hitachi Corporation, Tokyo, Japan) and 3.5 MHz probe (Hitachi EUP-C314T, Hitachi Corporation, Tokyo, Japan) with participants in the supine position at a central location in each village. The ultrasonographer was blind to infection status.

Liver ultrasonography was conducted according to the 1990 draft guidelines [13], [14]. Liver parenchymal fibrosis was graded 1 through 3 based on observed lesions, or 0 if none were present. Periportal fibrosis was assessed by grading the average diameter, from outer wall to outer wall, of three peripheral branches of the portal vein between the first and third branching point (grade 0: <3mm; grade 1: 3 to 5 mm; grade 2: >5 to 7 mm; grade 3: >7 mm). As done previously, grades 0 and 1 were combined for analysis [16]. The internal diameter of the portal vein was measured at the entry point of the portal vein into the liver. The length of the left liver lobe was measured in a longitudinal section along the left parasternal line, and the length of the right liver lobe was measured as the maximum oblique diameter using a right anterior axillary view according to the revised guidelines established for *S. mansoni* [15].

Two measurements of the spleen that are part of the standard Chinese examination were also included: spleen thickness, measured from the hilum to the opposite section, and the internal diameter of the spleen vein, measured at the entry point to the spleen [24].

Because organ and vein sizes vary with height, left and right liver lobe length, portal vein diameter and spleen thickness were evaluated using height-specific standard sizes drawn from a Chinese population where schistosomiasis is not endemic [18]. Measurements greater than two standard deviations above the mean size for height were classified as abnormal. Height was measured in 2002 in 440 of the 578 cohort members. The 343 adults (≥18 years old in 2000) with height measurements were assigned their 2002 height throughout the study. Children (<18 years old in 2000) were measured again in 2007 as part of a study described elsewhere [19]. The height measurements from 2002 and 2007 from 119 children (60 with 1 height measurement, 59 with two height measurements) were used to generate an age- and sex-dependent random intercept model in order to impute heights during the years children weren't measured. Height for child *i* at time *j* was calculated using the following equation:
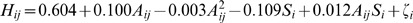
where *A_ij_* represents a child's age at time *j*, *S_i_* represents his or her sex (*S* = 1 for males) and 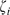 represents his or her random intercept. The model was fit using xtmixed in Stata 10.1 (StataCorp, College Station, TX, USA) and 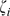 was predicted using empirical Bayes [25]. As random intercept models assume parametric distribution of residuals, first and second order residuals were examined and were normally distributed. The selected model was superior to a model that did not include a random intercept (likelihood ratio test, χ^2^ = 38.20, 1 d.f., p<0.001). Organ and vessel measures could not be height adjusted for the 86 adults and 26 children without height measurements. Participants with any versus no height data did not differ significantly by any of the predictors examined in the analyses including sex, mean age or infection status. No height standardized values were available for spleen vein diameter, so it was evaluated by the standard threshold used in China: >8 mm [24].

### Infection and treatment

Participants were tested for infection with *S. japonicum* in the fall of 2000 and 2002 using the miracidial hatch test [26] and the Kato-Katz thick smear procedure [27]. For each hatch test, a stool sample weighing at least 30 g was suspended in aqueous solution, filtered using copper mesh to remove large particles (40–60 mesh) followed by nylon gauze (260 mesh) to concentrate schistosome eggs. This sediment was re-suspended with distilled water in a 250 ml Erlenmeyer flask. Flasks were examined for miracidia 30–60 minutes, 4 hours and 8 hours after suspension if temperatures were above 30 degrees Celsius, or at 6, 12 and 18 hours at lower ambient temperatures. In 2000, three miracidial hatch tests were conducted per person using stool samples from three distinct days. In 2002, due to logistical constraints, one miracidial hatch test was conducted per person; however the Kato-Katz protocol was identical both years. The Kato-Katz procedure involved the preparation of three 41.5 mg slides from one homogenized stool sample in 2000 and 2002. Infection intensity, in eggs per gram of stool (EPG), was calculated as the total number of *S. japonicum* eggs present on the slides divided by the total sample weight. Participants were classified as infected if at least one test was positive. Everyone testing positive for *S. japonicum* was provided praziquantel treatment by the county Anti-Schistosomiasis Control Station. In addition, praziquantel was administered to all residents in the study villages in 2003, as part of a nation-wide effort to control infectious diseases following the outbreak of severe acute respiratory syndrome.

Participant age, sex, occupation and highest level of schooling were obtained by interview in fall 2000.

### Statistical analysis

In order to assess whether the participants in the ultrasound cohort were representative of the village populations from which they were selected, cohort participants were compared to individuals who participated only in the cross-sectional demographic and infection surveys in terms of age, sex, occupation, educational attainment and 2000 *S. japonicum* infection status. Similarly, cohort members with complete vs. incomplete follow-up were compared in terms of age, sex, baseline morbidity and infection in order to assess non-differential loss to follow-up. In both cases, comparisons were conducted using the *χ^2^* and t-test.

Analyses that included multiple observations from individuals accounted for within subject correlation of outcomes. Liver parenchymal fibrosis grade, an ordinal measure, was examined using ordinal logistic regression, a population averaged model using a sandwich type estimator for inference accounting for within-subject residual correlation [28]. Because ordinal logistic regression assumes the effect of a predictor on an outcome is constant for each stepwise increase in the outcome, the Brant test [29] was used to check that this parallel regression assumption was not violated. For all other liver and spleen measures and for predictors of *S. japonicum* infection status, generalized estimating equation (GEE) logistic regression with exchangeable correlation was conducted [30]. The Huber/White/sandwich estimator of variance was used which is robust to misspecification of the outcome distribution [31], [32].

The relationship between *S. japonicum* infection and ultrasound-detected abnormalities was first assessed by examining the impact of current infection status and, separately, infection intensity, on current ultrasound measures. Because infection intensity was highly right skewed, it was categorized into approximate quartiles among those who were infected. In order to examine the role of past infection on current morbidity, we also examined the relationship between infection status and intensity two to three years prior to the ultrasound examination and ultrasound-detected abnormalities (for example: 2000 infection status as a predictor of ultrasound-detected morbidity in 2002, and 2002 infection status as a predictor of ultrasound-detected morbidity in 2005). We hypothesized that age, sex and year of examination could modify the effect of infection on morbidity, so each model was run including all possible first-order interaction terms. The Wald test was used to test for significant interactions and if present, terms were removed from the model step-wise until only interaction terms significant at p-values <0.05 remained. In the absence of effect modification, these same variables could confound the relationship between infection and ultrasound-detected morbidity. While occupation, village and educational status were considered potential predictors of *S. japonicum* infection, they are unlikely to effect ultrasound morbidity independent of infection status. As they do not fit the definition of confounders [33], they were not controlled for in models examining the relationship between infection and ultrasound-detected abnormalities. The change in ultrasound-detected abnormalities over time was examined adjusting for age and sex.

We modeled age as a categorical variable when examining the relationship between age and ultrasound-detected morbidity to allow a non-linear relationship. Because liver abnormalities increase with age, age was treated as a continuous variable when included as a confounder in models testing the relationship between infection and morbidity, or changes in morbidity over time. Periportal fibrosis grade was modeled as a binary variable, as grades 0 and 1 were combined and no grade 3 fibrosis was detected. Tests for trend were calculated by treating categorical variables as ordinal. All results were assessed for statistical significance setting α = 0.05. Statistical analyses were conducted using Stata 10.1 (StataCorp, College Station, TX, USA).

## Results

In 2000, 578 people from ten villages were examined using ultrasound. The mean age of participants was 29.8 years (range 4–61 years). Half (51%) were female (Table 1). Most adults (≥18 years) were farmers (91%) and had no formal schooling beyond elementary school (59%). Participants were similar in terms of occupation, educational attainment and 2000 infection status to the 1,333 residents in these villages who participated in infection and demographic surveys only, although cohort members were slightly older than the rest of the population (mean age 29.8 vs. 28.2, p = 0.037).

**Table 1:** Characteristics of the 578 participants in the ultrasound cohort in 2000.

	No.	(%)
Sex		
Female	295	(51)
Male	279	(49)
Age		
<18	145	(25)
18–29	141	(25)
30–39	130	(23)
40–49	95	(17)
50+	63	(11)
Occupation^1^		
Not farmer	37	(9)
Farmer	392	(91)
Education^1^		
Elementary school or less	253	(59)
Some middle school or higher	175	(41)

1 Includes adults (>18 years) only.

We conducted ultrasound examinations with 444 people in 2002 and 321 people in 2005. The 320 people with complete ultrasound follow-up were no more likely to be infected with *S. japonicum* at enrollment than those who missed at least one follow-up examination, but they were older (mean age 33.0 vs. 25.6, p<0.001) and more likely to have at least one liver abnormality in 2000 (57% vs. 37%, p<0.001).

Nearly half (47%) of participants tested positive for *S. japonicum* in 2000. Mean infection intensity was 53.4 EPG. In 2002, infection prevalence declined to 32% and intensity to 9.4 EPG. As shown in Table 2, adults aged 50 and older were less likely to be infected than younger participants. Neither sex nor occupation was associated with infection prevalence, but among adults, higher educational attainment was protective. Infection prevalence in 2002 was significantly higher among those who were infected vs. uninfected in 2000 despite the distribution of treatment to everyone who tested positive (41% vs. 23%, p<0.001). Infection prevalence varied significantly by village.

**Table 2:** The distribution of *S. japonicum* infection by age, sex, occupation, educational attainment, year and village.

	2000			2002			
	n	Infected (%)		n	Infected (%)		OR (95% CI)^1^
Year							
Year 2000	564	265	(47)				1.00
Year 2002				504	161	(32)	0.53 (0.42–0.66)
Sex							
Female	288	147	(51)	260	78	(30)	1.00
Male	276	118	(43)	244	83	(34)	0.89 (0.69–1.16)
Age							
<18	138	63	(46)	101	29	(29)	1.00
18–29	140	65	(46)	98	30	(31)	1.06 (0.72–1.56)
30–39	129	70	(54)	150	59	(39)	1.33 (0.93–1.92)
40–49	95	49	(52)	75	23	(31)	1.16 (0.78–1.73)
50+	62	18	(29)	80	20	(25)	0.58 (0.36–0.95)
p-value, test for trend^2^							0.209
Occupation^3^							
Not farmer	37	13	(35)	35	9	(26)	1.00
Farmer	389	189	(49)	368	123	(33)	1.59 (0.84–3.01)
Education^3^							
Elementary school or less	252	134	(53)	240	81	(34)	1.00
Some middle school or higher	173	68	(39)	162	51	(31)	0.70 (0.51–0.96)
Village							
Xinming3	63	32	(51)	54	18	(33)	1.00
Tuanjie2	58	6	(10)	56	8	(14)	0.19 (0.09–0.39)
Jiaojia4	59	13	(22)	54	7	(13)	0.29 (0.15–0.54)
Hexing1	61	19	(31)	59	8	(14)	0.39 (0.22–0.71)
Minhe1	67	29	(43)	55	23	(42)	0.99 (0.57–1.72)
Minhe3	61	35	(57)	56	20	(36)	1.18 (0.71–1.99)
Xinlong7	45	27	(60)	38	16	(42)	1.45 (0.80–2.62)
Jianxing6	60	41	(68)	48	21	(44)	1.81 (1.09–3.03)
Xinming7	45	31	(69)	42	19	(45)	1.82 (1.06–3.11)
Shian5	45	32	(71)	42	21	(50)	2.08 (1.14–3.81)

1 Odds ratios account for multiple observations from the same individual using generalized estimating equations with exchangeable correlation and robust inference.

2 Test for trend was calculated by treating age categories as ordinal.

3 Analysis included adults (≥18 years) only.

Table 3 describes the prevalence of liver parenchymal fibrosis, periportal fibrosis and abnormal liver and spleen measurements from 2000 to 2005. Following the initiation of schistosomiasis testing and treatment of all infections in 2000, parenchymal fibrosis, periportal fibrosis and right liver lobe enlargement decreased significantly through 2005, controlling for age and sex (Table 4). Decreases in spleen enlargement were also observed, although the trend was of marginal significance (p = 0.091).

**Table 3:** Prevalence of abnormal liver and spleen measurements from 2000 to 2005.

	2000		2002		2005	
	No.	(%)	No.	(%)	No.	(%)
Parenchymal fibrosis						
Grade 0	388	(67)	297	(67)	219	(68)
Grade 1	83	(14)	50	(11)	35	(11)
Grade 2	92	(16)	88	(20)	58	(18)
Grade 3	15	(3)	9	(2)	9	(3)
Periportal fibrosis						
Grade 0 (av. diameter <3 mm)	22	(4)	24	(5)	19	(6)
Grade 1 (av. diameter 3 to 5 mm)	535	(93)	401	(90)	299	(93)
Grade 2 (av. diameter 5.1 to 7 mm)	21	(4)	19	(4)	3	(1)
Grade 3 (av. diameter >7 mm)	0	(0)	0	(0)	0	(0)
Left liver lobe^1^						
Normal	389	(84)	373	(85)	250	(79)
Enlarged	73	(16)	67	(15)	67	(21)
Right liver lobe^1^						
Normal	351	(76)	402	(91)	277	(87)
Enlarged	111	(24)	38	(9)	40	(13)
Portal vein^1^						
Normal	417	(90)	383	(87)	274	(86)
Dilated	45	(10)	57	(13)	43	(14)
Spleen (thickness)^1^						
Normal	442	(96)	425	(97)	310	(98)
Enlarged	18	(4)	14	(3)	6	(2)
Spleen vein^2^						
Normal	549	(95)	406	(92)	313	(98)
Dilated	28	(5)	37	(8)	7	(2)

1 Enlargement of the left liver lobe, right liver lobe and spleen, and portal vein dilation were classified based on height-specific normal values [18].

2 The spleen vein was classified as dilated if the diameter exceeded 8 mm.

**Table 4:** The relationship between ultrasound-detected abnormalities and age, sex and year.

	Parenchymal fibrosis		Periportal fibrosis^1^		Left liver lobe enlargement^2^		Right liver lobe enlargement^2^		Portal vein dilation^2^		Spleen enlargement^2^		Spleen vein dilation	
	OR (95% CI)		OR (95% CI)		OR (95% CI)		OR (95% CI)		OR (95% CI)		OR (95% CI)		OR (95% CI)	
Sex														
Female	1.00		1.00		1.00		1.00		1.00		1.00		1.00	
Male	1.18	(0.85–1.64)	4.35	(2.05–9.23)	0.41	(0.28–0.60)	1.88	(1.29–2.73)	1.28	(0.87–1.89)	0.89	(0.43–1.83)	1.22	(0.74–2.01)
Age^3^														
<18	1.00		1.00		1.00		1.00		1.00		1.00		1.00	
18–29	10.44	(5.11–21.33)	9.36	(1.14–76.56)	2.91	(1.35–6.29)	2.40	(1.25–4.61)	2.00	(0.93–4.33)	3.40	(1.02–11.34)	3.18	(1.34–7.58)
30–39	11.76	(5.96–23.18)	9.79	(1.31–73.01)	4.00	(2.03–7.89)	2.91	(1.61–5.28)	3.19	(1.58–6.44)	2.09	(0.63–6.88)	2.60	(1.11–6.12)
40–49	30.76	(15.21–62.20)	10.95	(1.41–85.30)	4.56	(2.25–9.23)	3.13	(1.65–5.95)	2.82	(1.35–5.91)	1.74	(0.45–6.78)	1.35	(0.46–3.94)
50+	35.89	(17.45–73.80)	8.47	(1.07–67.07)	6.21	(3.05–12.63)	3.26	(1.66–6.41)	3.98	(1.97–8.06)	1.82	(0.47–6.98)	2.32	(0.93–5.79)
trend^4^	<0.001		0.025		<0.001		<0.001		<0.001		0.894		0.548	
Year^5^														
2000	1.00		1.00		1.00		1.00		1.00		1.00		1.00	
2002	0.82	(0.68–0.98)	1.07	(0.59–1.96)	0.86	(0.62–1.19)	0.26	(0.18–0.37)	1.28	(0.87–1.88)	0.79	(0.42–1.49)	1.79	(1.09–2.93)
2005	0.57	(0.44–0.73)	0.20	(0.06–0.63)	1.11	(0.77–1.60)	0.35	(0.24–0.52)	1.20	(0.78–1.84)	0.43	(0.16–1.20)	0.41	(0.17–0.95)
trend^4^	<0.001		0.002		0.634		<0.001		0.362		0.091		0.119	

1 Periportal fibrosis was treated as a binary variable, comparing grade 2 to grades 0 and 1.

2 Enlargement of the left liver lobe, right liver lobe, spleen, and portal vein dilation were classified based on height-specific normal values.

3 There were no children (<18) with grade 3 parenchymal fibrosis, leading to a violation of the parallel regression assumption for ordinal logistic regression. For this analysis, grade 3 was grouped with grade 2 in order to avoid a model violation.

4 The test for trend was calculated by treating categories as ordinal.

5 Analysis adjusted for age and sex.

All odds ratios account for multiple observations from the same individual. Ordinal logistic regression with a sandwich type estimator was used for parenchymal fibrosis. Generalized estimating equations logistic regression with exchangeable correlation was used for all other measures.

Liver abnormalities increased significantly with age, most notably for parenchymal fibrosis (Table 4). Spleen enlargement was not associated with age. Individuals aged 18 to 29 years had the highest odds of spleen vein dilation. The relationship between liver abnormalities and sex varied: men were more likely to have periportal fibrosis and enlarged right liver lobes; women were more likely to have enlarged left liver lobes.

Schistosomiasis infection at the time of ultrasound was associated with an increase in liver parenchymal fibrosis grade (OR 1.40, 95% CI: 1.03–1.90) adjusting for age, sex and year of ultrasound (Table 5). Infection intensity at the time of ultrasound was also associated with an increase in liver parenchymal fibrosis grade (test for trend: p = 0.002). Individuals with greater than 50 EPG had 2.10 times greater odds of more advanced fibrosis than those not excreting eggs (95% CI: 1.33–3.32).

**Table 5:** The impact *S. japonicum* infection on ultrasound-detected liver abnormalities.

	Parenchymal fibrosis				Left liver lobe enlargement				Portal vein dilation			
	Adjusted^1^		Unadjusted		Adjusted^1^		Unadjusted		Adjusted^1^		Unadjusted	
	OR	(95% CI)	OR	(95% CI)	OR	(95% CI)	OR	(95% CI)	OR	(95% CI)	OR	(95% CI)
I. Current infection (n = 977)^2^												
Uninfected	1.00		1.00		1.00		1.00		1.00		1.00	
Infected	1.40	(1.03–1.90)	1.21	(0.91–1.61)	1.05	(0.73–1.51)	1.00	(0.72–1.40)	0.86	(0.55–1.35)	0.80	(0.51–1.25)
II. Current inf. intensity (n = 976)^2^												
0 EPG	1.00		1.00		1.00		1.00		1.00		1.00	
1–10 EPG	1.50	(0.99–2.27)	1.36	(0.91–2.05)	1.04	(0.55–1.97)	1.02	(0.55–1.91)	1.10	(0.56–2.15)	1.05	(0.54–2.04)
11–50 EPG	1.30	(0.82–2.08)	1.22	(0.81–1.85)	0.94	(0.50–1.75)	0.93	(0.51–1.69)	0.94	(0.45–1.98)	0.86	(0.40–1.81)
>50 EPG	2.10	(1.33–3.32)	1.46	(0.95–2.25)	1.71	(0.94–3.12)	1.47	(0.84–2.55)	0.82	(0.35–1.93)	0.66	(0.28–1.57)
p-value, test for trend^3^	0.002		0.052		0.185		0.301		0.691		0.353	
III. Prior infection (n = 739)^4^												
Uninfected	1.00		1.00		1.00		1.00		1.00		1.00	
Infected	1.84	(1.30–2.60)	1.48	(1.06–2.05)	1.45	(0.97–2.17)	1.18	(0.81–1.72)	1.09	(0.71–1.68)	1.00	(0.65–1.52)
IV. Prior inf. intensity (n = 739)^4^												
0 EPG	1.00		1.00		1.00		1.00		1.00		1.00	
1–10 EPG	1.71	(1.04–2.79)	1.42	(0.90–2.24)	1.21	(0.68–2.15)	1.07	(0.62–1.86)	0.50	(0.20–1.29)	0.47	(0.19–1.17)
11–50 EPG	2.07	(1.21–3.57)	1.79	(1.08–2.97)	1.39	(0.79–2.42)	1.17	(0.67–2.04)	1.37	(0.71–2.66)	1.25	(0.66–2.38)
>50 EPG	2.84	(1.71–4.73)	1.81	(1.14–2.89)	1.12	(0.57–2.20)	0.79	(0.41–1.52)	2.02	(1.05–3.89)	1.55	(0.84–2.85)
p-value, test for trend^3^	<0.001		0.002		0.440		0.712		0.051		0.194	

1 Adjusted models controlled for age, sex and year of ultrasound.

2 Current infection status and intensity was assessed at the time of an ultrasound examination.

3 The test for trend was calculated by treating infection intensity categories as ordinal.

4 Prior infection status and intensity was assessed two to three years before an ultrasound examination (in 2000 for the 2002 ultrasound examination, 2002 for the 2005 ultrasound examination).

All odds ratios account for multiple observations from the same individual. Ordinal logistic regression with a sandwich type estimator was used for parenchymal fibrosis. Generalized estimating equations logistic regression with exchangeable correlation was used for all other measures.

For each liver abnormality, four measures of infection are examined: current infection status, current infection intensity, prior infection status and prior infection intensity.

Schistosomiasis infection two to three years prior to ultrasound was associated with an increase in liver parenchymal fibrosis grade, and the association was stronger than that of current infection (OR 1.84, 95% CI: 1.30–2.60). Prior infection intensity was also associated with liver parenchymal fibrosis (test for trend, p<0.001). Individuals with greater than 50 EPG two to three years prior to the ultrasound examination had 2.84 times greater odds of advanced fibrosis than those not excreting eggs two to three years prior to ultrasound (95% CI: 1.71–4.73).

The other hepatosplenic ultrasound measures were not associated with current infection status or intensity. Several measures were associated with prior infection, although the relationships were not as consistent as those observed for periportal fibrosis. Prior infection appeared to elevate the probability of left liver lobe enlargement (OR 1.45, 95% CI: 0.97–2.17). Prior infection intensity but not prior infection status, was associated with increased odds of portal vein dilation (test for trend, p = 0.051). The impact of prior infection status on right liver lobe enlargement varied by the year of examination and the sex of the participant. Prior infection was associated with increased odds of right liver lobe enlargement among males examined in 2005 (OR 3.95, 95% CI: 1.82–8.57) and decreased odds of right liver lobe enlargement among females examined in 2002 (OR 0.33, 95% CI: 0.13–0.86). Prior infection intensity was not associated with right or left liver lobe enlargement. Due to the limited number of individuals with periportal fibrosis, models were unable to yield stable estimates of the effect of prior infection on this measure Spleen enlargement and spleen vein dilation were not associated with current or prior infection.

Most people with liver and spleen enlargement, portal vein dilation, periportal fibrosis or spleen vein dilation in 2000 had normal pathology by 2002 (Table 6). However, this was not the case for parenchymal fibrosis: 67% of people with grade 2 fibrosis and 100% with grade 3 fibrosis in 2000 remained at or above grade 2 throughout the five-year follow-up.

**Table 6:** The reversal of ultrasound-detected liver and spleen abnormalities among individuals with abnormal measures in 2000.

	No. in 2000^1^	Reversed in 2002		Reversed in 2005		Never reversed	
		No.	(%)	No.	(%)	No.	(%)
Parenchymal fibrosis (grade 2 or 3)^2^	71	11	(15)	17	(24)	51	(72)
Periportal fibrosis (grade 2)	17	14	(82)	16	(94)	0	(0)
Left liver lobe enlarged	58	35	(60)	35	(60)	13	(22)
Right liver lobe enlarged	85	65	(76)	63	(74)	10	(12)
Portal vein dilated	33	22	(67)	24	(73)	5	(15)
Spleen enlarged	16	13	(81)	16	(100)	0	(0)
Spleen vein dilated	16	12	(75)	15	(94)	0	(0)

1 Includes only individuals with complete follow-up data.

2 Regression to parenchymal fibrosis grade 0 or 1 was classified as reversal.

## Discussion

We found evidence of a direct, exposure-response relationship between *S. japonicum* infection and parenchymal fibrosis. While there has been suggestive evidence of an association between infection and parenchymal fibrosis, including an association between progression of parenchymal fibrosis and current infection [16], this is the first study to show the risk of parenchymal fibrosis is higher in people who are infected vs. uninfected, and highest in individuals with the greatest infection intensities. Parenchymal fibrosis declined significantly following treatment however, improvements were limited among individuals with advanced fibrosis: 72% of people with severe fibrosis at enrollment (grades 2 or 3) had not resolved below grade 2 by the end of the five-year study. This suggests parenchymal fibrosis is an appropriate measure of *S. japonicum* morbidity and can document improvements in morbidity following treatment, although little improvement may be observed among those with advanced fibrosis.

In contrast, the remaining measures were less consistently associated with *S. japonicum* infection and are of questionable epidemiological use in regions with similar infection levels. Periportal fibrosis was rare in this population and could not be associated with *S. japonicum* infection, although it did decline significantly following the initiation of targeted treatment. Others have used image-based classification to assess periportal fibrosis [34], [35], which has been shown to have better reproducibility for *S. mansoni*-related fibrosis, perhaps because it does not require the ultrasonographer to measure narrow vessel widths [36]. Our measures of periportal fibrosis were not height adjusted, as Chinese standards for the diameter of portal vein branches as measured here have not been published. The lack of height adjustment may have led to underestimates of the prevalence of periportal fibrosis and may explain the higher prevalence of periportal fibrosis in men, however higher prevalence of fibrosis has also been observed in males using image-based classification [35].

The remaining three hepatic measures, left and right liver lobe enlargement and portal vein dilation, were associated with *S. japonicum* infection or declined following chemotherapy. However, the relationships between *S. japonicum* infection and these morbidity measures were less consistent than those observed with parenchymal fibrosis and several associations were of marginal statistical significance. The spleen measures were neither associated with infection nor did they decline with treatment. All five measures require the ultrasonographer to measure organ or vessel sizes and participant's height. Like the measures of periportal fibrosis used here, these methods present opportunities for measurement error which may bias estimates of exposure-disease relationships toward the null, producing attenuation and exacerbating non-linearity [37]. The reproducibility of some of these measures has been shown to be poor in a pediatric population, and further efforts to evaluate the accuracy of organ and vein size measurements are needed [19].

Overall, the prevalence of morbidity in this cohort was lower than has been observed elsewhere in China as was infection intensity [16], [17], [34], [38]. Measures of liver and spleen enlargement, portal vein dilation and spleen vein dilation were less informative than measures of parenchymal fibrosis but may be appropriate in areas where infection intensity and associated morbidities are higher. Declines in hepatomegaly and splenomegaly have been demonstrated following treatment in regions with higher average worm burdens [16], [17]. The strong associations between age and all liver measures highlight the importance of considering age as a potential confounder when examining the relationship between *S. japonicum* infection and morbidity and declines in morbidity over time. For example, the unadjusted prevalence of parenchymal fibrosis did not change over time, however as loss to follow-up was highest in younger populations which were least likely to have fibrosis, the age and sex adjusted prevalence did decline.

Our findings shed light on the development of hepatic fibrosis following *S. japonicum* infection. The lack of reversal of grade 3 parenchymal fibrosis to grade 1 or normal and the association between past infection and current fibrosis suggest severe fibrosis may persist or even progress following treatment. Prior studies have also found minimal reversal of severe fibrosis following treatment [16], [39] and hepatic fibrosis has been documented in people living in areas where the parasite, and therefore exposure, was eliminated 20 years previously [40]. While treatment with praziquantel can reduce infection prevalence, intensity and fibrosis, this analysis provides further evidence that severe hepatic fibrosis may be unlikely to reverse quickly. Minimal declines in severe hepatic fibrosis associated with *S. mansoni* infection have also been detected [41] but other studies have noted complete reversal of severe morbidity [42].

Further, parenchymal fibrosis was associated with current infection status and intensity but more strongly associated with infection status and intensity two to three years prior to ultrasound, despite timely treatment of infections. The surprising association between past infection and fibrosis raises questions about the progression of fibrosis following infection and treatment. Praziquantel kills the adult worm but schistosome eggs can remain trapped in host tissues decades after exposure [43]. While schistosome eggs are thought to disintegrate within weeks of granuloma formation [7], it is possible, due to an inflammatory cascade, fibrogenesis continues over a longer time period. Collagen deposition has been shown to occur following treatment with praziquantel in *S. japonicum* infected mice, suggesting fibrosis may continue despite removal of adult worms [44]. In humans, progression of fibrosis has been observed following treatment and was not explained by reinfection [21], [45]. Alternatively, it is possible that the relationship between past infection and fibrosis is due to reinfection. Past infection predicted subsequent infection. However, if reinfection rather than past infection determined fibrosis, one would expect stronger relationships between current infection and fibrosis than between past infection and fibrosis, which was not observed. It is also possible that some individuals who were treated were not cured. A single dose of praziquantel cures 90% of *S. japonicum* infections [46], suggesting 10% of those treated may continue to harbor adult worms and face continued egg production. In our work in this region, we have also found individuals who decline to take praziquantel even after a positive infection test. Non-compliance and treatment failure are realities of any chemotherapy-based control program, however the marked declines in infection prevalence, intensity and morbidity suggest the number of individuals who were not cured was limited.

This study examined one of the largest populations to be followed over five years in order to assess ultrasound-detectable morbidity and *S. japonicum* infection. Participants were randomly sampled from a comprehensive cross-sectional survey in order to minimize selection bias. Loss to follow-up was greater among those without ultrasound-detectable morbidity, which suggests the prevalence of morbidity in 2002 and 2005 may be overestimated, and therefore the true declines in morbidity over time may be greater than observed. Retention was independent of infection status, minimizing bias in estimates of the impact of infection on pathology. No information was available on alcohol consumption or infection with hepatitis B virus (HBV), two factors that can induce liver pathology and may exacerbate schistosomiasis morbidity. The parenchymal network pattern due to *S. japonicum* is distinct from the lesions produced by HBV, as HBV produces a finer, meshwork texture [47], suggesting the observed prevalence of parenchymal fibrosis is specific to schistosomiasis. While HBV has been shown to hinder regression of periportal fibrosis following treatment of *S. mansoni* infections [41], the extent to which HBV exacerbates morbidity due to *S. japonicum* remains unclear and warrants further study. Reductions in parenchymal fibrosis may be impaired by alcohol consumption, which in our study areas is confined almost exclusively to males [17]. Unless alcohol consumption or HBV impacts a person's probability of infection, they are unlikely to confound the relationships between infection and ultrasound-detected morbidity.

Community-wide testing and treatment of all infections with praziquantel yielded marked declines in infection prevalence and intensity. However, reinfection was high: 32% of people were infected two years after the first round of targeted treatment. High rates of infection following treatment are not uncommon and underscore the challenges of sustainably reducing human schistosomiasis [48]. Adults aged 50 and over were less likely to be infected than younger populations, possibly corresponding to a decline in water contact later in life or acquired immunity [49].

In summary, we present evidence that ultrasound-detectable liver fibrosis is associated with *S. japonicum* infection status and intensity, controlling for age, sex and year of ultrasound examination, and this measure can be used to monitor *S. japonicum* induced morbidity. Other ultrasound measures including hepatomegaly and splenomegaly were less clearly associated with infection. Our findings also suggest that some morbidity may not reverse within five years of treatment and may even progress despite treatment. Praziquantel has yielded remarkable declines in schistosomiasis morbidity in China and throughout the globe but reinfection following treatment is common as observed in this study population. *S. japonicum* infection, particularly high egg loads, may lead to fibrosis that is not rapidly reversed by treatment, underscoring the importance of measures to prevent new infections as well as treating current disease.

## Supporting Information

Checklist S1 STROBE checklist.
(0.09 MB DOC)

Alternative Language Abstract S1 Translation of abstract into Chinese by YZ.
(0.03 MB DOC)
