# Supplementary material for: The Impact of Schistosoma japonicum Infection and Treatment on Ultrasound-Detectable Morbidity: A Five-Year Cohort Study in Southwest China
Source: PLoS Negl Trop Dis. 2010 May 18;4(5):e685. doi: 10.1371/journal.pntd.0000685 (PMC2872638; doi:10.1371/journal.pntd.0000685)
Supplement: Alternative Language Abstract S1 — Translation of abstract into Chinese by YZ. (0.03 MB DOC) [file pntd.0000685.s002.doc]

**日本血吸虫感染和治疗对超声可见病变的影响：一项在中国西南地区为期五年的队列研究**

**页首标题：**日本血吸虫与超声可见病变

**摘要**

***背景：***超声波扫描提供了对肝脏和脾脏的非侵入性检查手段，并能够增加研究者对血吸虫病变的了解。

***方法/主要发现：***研究者对居住在中国西南地区的578人进行了为期5年的追踪；通过粪检确定参与者的日本血吸虫病感染状况，并利用超声波对参与者的肝脏及脾脏进行了七项常规检测，以评估血吸虫感染与超声波可见病变的联系，以及针对性治疗对病变的影响。研究发现，调整年龄、性别和年份等因素后，管壁纤维化，即一种由日本血吸虫造成的肝脏网状结构特征，与参与者进行超声波检测时的感染状况（OR 1.40, 95% CI: 1.03 - 1.90）及感染强度（趋势检验p=0.002）相关；并与参与者以往的感染状况和感染强度有更强的相关性（调整后OR 1.84, 95% CI: 1.30 - 2.60; 趋势检验p<0.001），即使对参与者进行了即时的治疗。此外，虽然管壁纤维化程度随时间的衰减有统计学意义，但在研究初始时纤维化严重（等级2或3）的参与者中，只有18%的人在随后的5年中转为等级1或正常。其它类型的肝脏异常则与日本血吸虫感染缺乏稳定的关联。

***结论/意义：***管壁纤维化是日本血吸虫病变的一项适宜的测量标准，并能够记录病情控制过程中疾病的转归情况。在感染程度相似的地区，其它的超声波检测项目的流行病学价值可能较为有限。由于严重的纤维化难以通过血吸虫病治疗迅速转归，因此对血吸虫病变的预防应在治疗的同时结合减少日本血吸虫暴露的控制措施。
